# Silicone granuloma mimicking Breast Implant Associated Large Cell Lymphoma (BIA-ALCL): a case report

**DOI:** 10.1080/23320885.2020.1762495

**Published:** 2020-05-23

**Authors:** Elizabeth Shepard, Srdjan Kamenko, Olivia L. Snir, Juliana Hansen

**Affiliations:** a School of Medicine, University of Washington, Seattle, WA, USA; b Division of Plastic and Reconstructive Surgery, Oregon Health and Sciences University, Portland, OR, USA; c Department of Pathology, Oregon Health and Science University, Portland, OR, USA

**Keywords:** Breast implant associated anaplastic large cell lymphoma (BIA-ALCL), silicone granuloma, mimicker, adenitis

## Abstract

We describe the case of a 75-year-old woman with textured silicone implants who was referred to our institution with concern for implant rupture and Breast Implant Associated Anaplastic Large Cell Lymphoma (BIA-ALCL). After explantation and pathologic evaluation, she was diagnosed with silicone granuloma and adenitis, though her presentation mimicked BIA-ALCL.

## Introduction

Breast Implant Associated Anaplastic Large Cell Lymphoma (BIA-ALCL) is a rare peripheral T cell lymphoma most commonly associated with textured breast implants [1–3]. It occurs 7–10 years after implantation and typically presents as either an isolated, rapidly accumulating seroma or as a discrete breast mass, with the latter being less common and conferring a worse prognosis [1,2,4]. This disease has captured the public’s attention as more than ten million women world-wide have breast prostheses [1]. As the fund of oncologic and epidemiologic research on BI-ALCL expands, so too does the effort to disperse this information to health care professionals, product manufacturers and the public [5–7].

While it is prudent to exclude BIA-ALCL as the cause of late seromas or new masses, it is important to remember that non-neoplastic diagnoses continue to remain a more likely explanation for the observed findings. For example, silicone granulomas due to implant rupture can mimic cancer both clinically and radiographically [8,9]. Additionally, late and unprovoked seroma formation may represent pathologic entities separate from lymphoma [10].

We describe the case of a woman with silicone granulomas from implant rupture, whose presentation mimicked that of BIA-ALCL. The goal of this case description is to emphasize the importance of maintaining a broad working differential diagnosis when evaluating patients with signs and symptoms concerning for BIA-ALCL. Doing so prevents unnecessary emotional distress for patients and families, and prevents a delay in diagnosis.

## Case description

**Figure 1.**
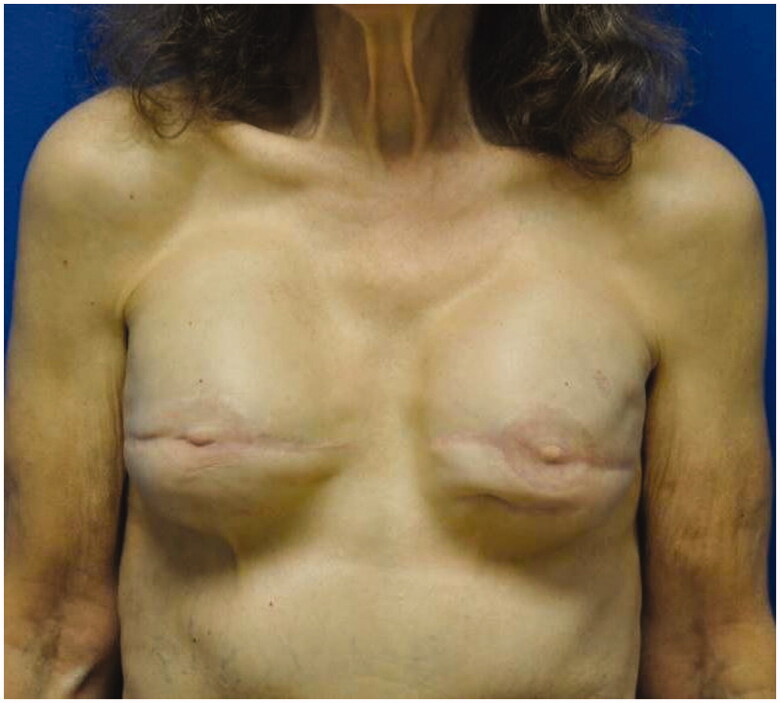
Preoperative photograph of the patient, taken anteriorly.

A 75-year-old woman with a remote history of bilateral invasive lobular breast cancer treated with skin sparing mastectomies, axillary node dissection and textured implant-based reconstruction in 1981 was referred to our clinic to discuss surgical treatment options in the setting of concern for BIA-ALCL. Following her initial surgery she developed two episodes of capsular contracture, seven and eleven years post-operatively, each requiring explantation and implant exchange. At the time of presentation her prostheses were 27-year-old sub-muscular, silicone, textured implants (Figure 1).

**Figure 2.**
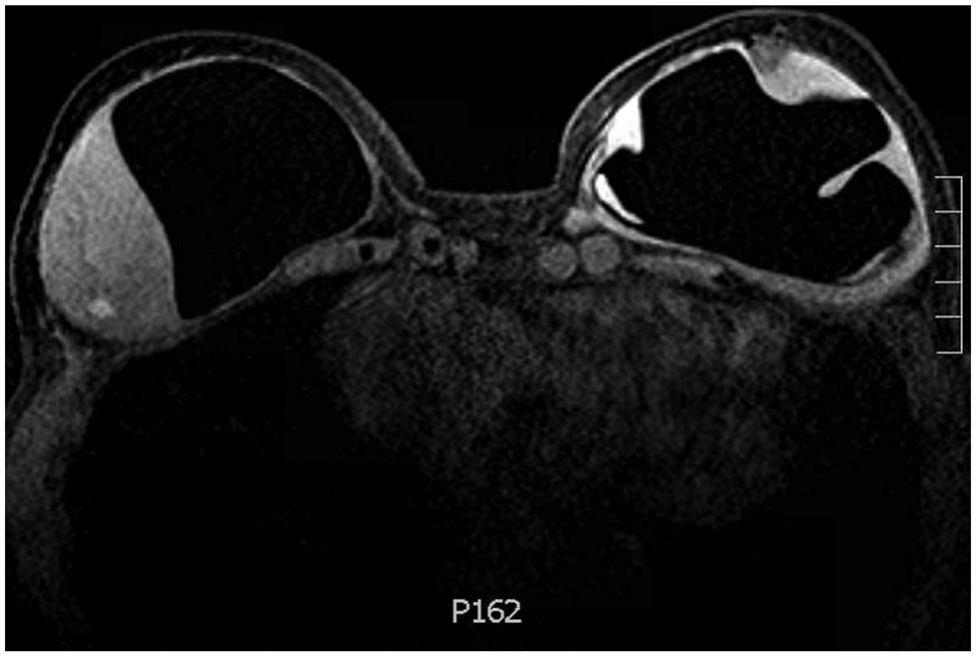
T1 weighted contrast bilateral breast MRI demonstrating a soft tissue intensity mass-like area attached to the right breast capsule with internal enhancing foci and trace periprosthetic fluid, in addition to enhancing foci at the deep margin of the left breast capsule.

She had no breast concerns until 6 months prior to presentation when she first noticed a painless, palpable, right-sided infraclavicular lump. The mass was located at the 1 o’clock position, 6–9 cm from the nipple. She denied the presence of overlying skin changes or systemic symptoms. She was evaluated by oncology and underwent a series of imaging studies. Magnetic resonance imaging (MRI) with contrast demonstrated right intracapsular and extracapsular rupture, an intracapsular enhancing mass-like area measuring 5.4 × 5.9 × 3.5 cm, trace peri-prosthetic fluid and multiple enhancing sub-centimeter foci distributed throughout the breast tissue. The left implant was intact and showed similarly enhancing foci at the lower outer quadrant along the capsule, in addition to a 15 mm fluid collection (Figure 2). Scattered lymph nodes measuring 4–12 mm were present throughout the superior mediastinum, internal mammary region, hilar region and left axilla. CT chest (with contrast) confirmed the breast and nodal findings. PET scan demonstrated very mild radiotracer uptake along the right chest wall mass (SUV 1.9), moderately intense uptake along the left lower outer breast quadrant and at the mediastinal and hilar notes (SUV 3.1–4.2), and intense uptake in the left axillary lymphadenopathy (SUV 8.7). Taken together, her presentation was suspicious for lymphoma and she was referred to plastic and reconstructive surgery clinic. Her exam was consistent with the above description and she was scheduled for bilateral exploration, breast implant removal, capsulectomy and implant replacement.

**Figure 3.**
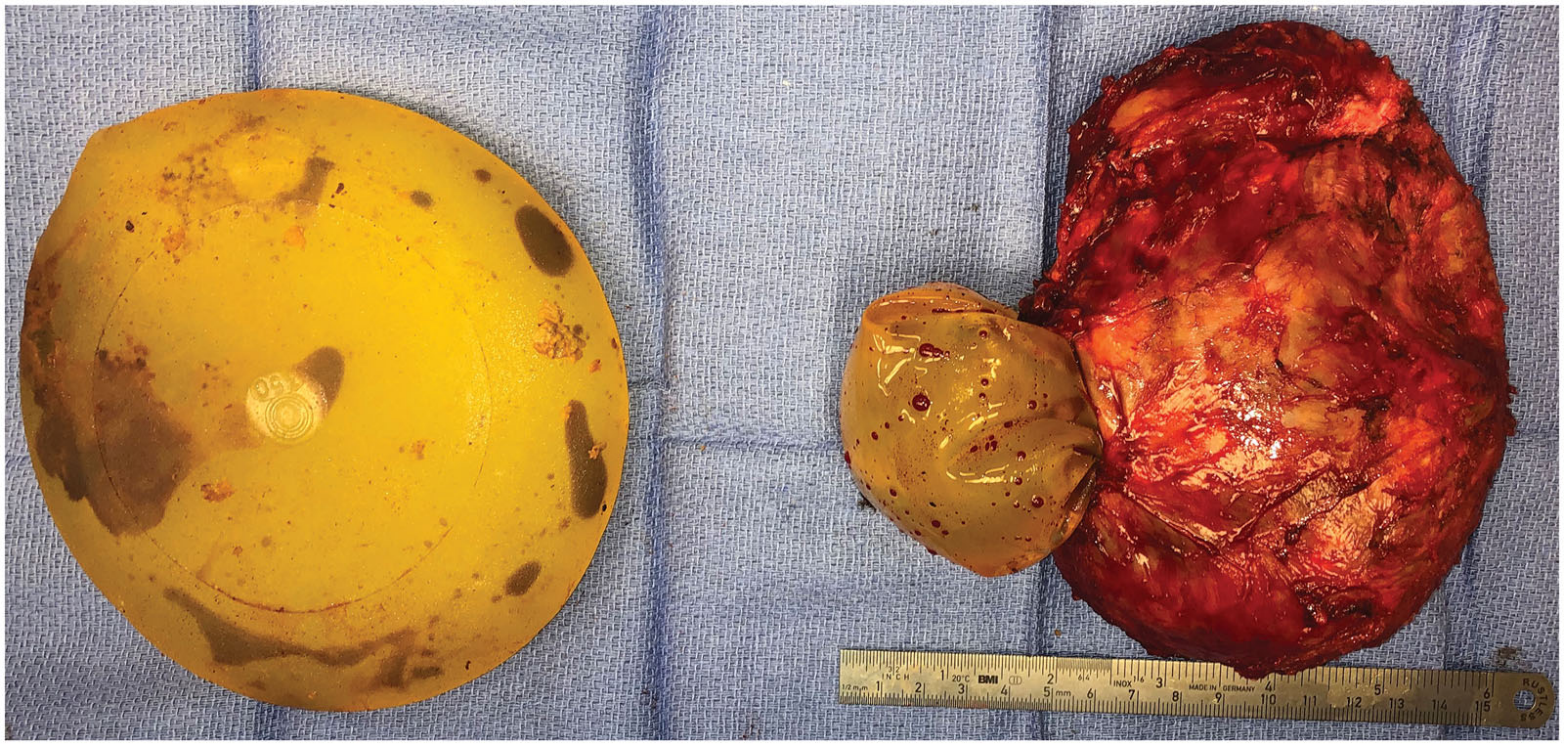
Intraoperative photograph of bilateral explanted, textured, silicone breast prostheses and right breast capsule. There is evidence of bilateral rupture with hematoma within the implants.

In the operating room the right implant was found to be extensively adhered to surrounding tissue and was removed in an en block fashion. Two masses were noted; one on the external surface which was homogenous, fleshy, tan-white colored and firm, and another on the internal surface of the hollow capsule which was heterogenous, hemorrhagic, yellow-tan colored and friable. The left implant was found to be ruptured (Figure 3). A subtotal capsulectomy was preformed, leaving behind some of the posterior capsule due to significant adhesions to the chest wall. The implant cavities were extensively irrigated and implants were replaced. She was seen for follow up at one, two and six weeks post-operative without concerns or complications.

**Figure 4.**
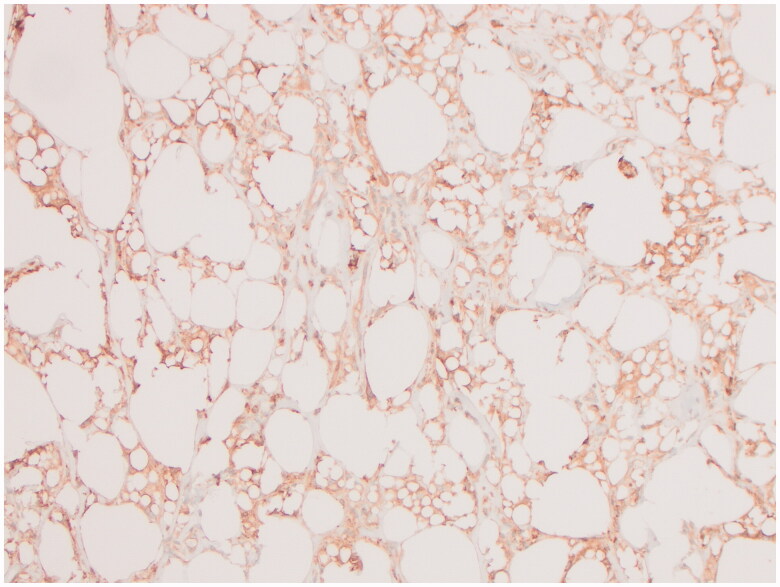
Immunohistochemistry for CD68, a histocyte marker, highlights numerous foam histocytes within the lesion on the external surface of the right capsule.

**Figure 5.**
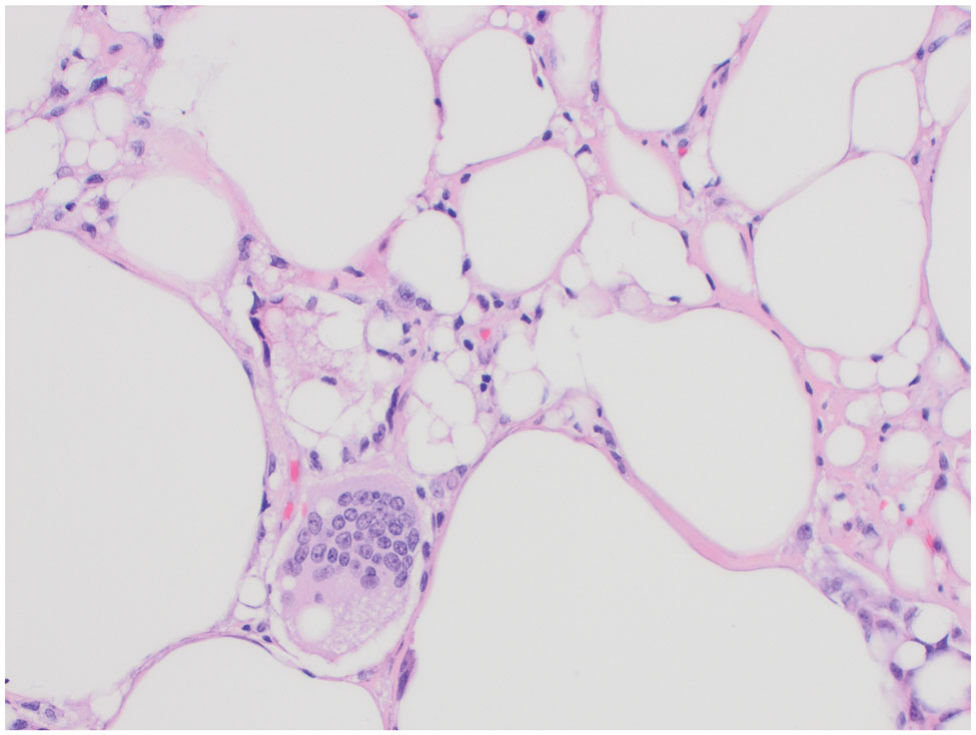
This image shows vacuolated histiocytes and a foreign body-type giant cell, features of the silicone granuloma taken from the lesion on the external surface of the right capsule.

Both breast specimens were negative for malignancy. Microscopic examination of the right external lesion showed abundant CD68 positive, CD30 negative histiocytes and silicone, while the internal lesion predominantly consisted of fibrin, hemorrhage and fibrous tissue. A portion of the lesion on the right external surface was submitted for flow cytometry and was inconsistent with BIA-ALCL. The left breast specimen also showed histocytes, mild chronic inflammation and free silicon. Her pathology was consistent with a silicone granuloma (Figures 4 and 5).

## Discussion

Silicone granulomas have been reported in the literature to mimic breast cancer, though none specifically address this mimicry in the context of BIA-ALCL. This is likely because the fund of knowledge surrounding BIA-ALCL has expanded rapidly in recent years. Since it was first described in the literature in 1997 the incidence has continued to climb; with current estimates ranging from 1:3817 to 1:30,000 in women who have received textured implants [2,10]. Our patient’s presentation was especially suspicious for BIA-ALCL given the age and texture of her implants, unprovoked seroma formation and the radiographic features of her breast masses and adenopathy. However, many of the signs, symptoms and risk factors for BIA-ALCL overlap with those of silicone granuloma.

The major risk factors for BIA-ALCL are the presence of textured implants and an increased age of the prosthesis. The risk factors for silicone implant rupture include increased patient age, sub-muscular pocket placement, as well as increased age of the prosthesis. Additionally, rates of rupture vary by manufacturer, model and indication; but typically ranges from 5% to 15% at 10 years post-operative [9,11]. Notably, textured implants have not been implicated as risk factor for implant rupture [9,12]. Our patient carried risk factors for both BIA-ALCL and implant rupture, though implant rupture with silicone granuloma formation was not included in the differential diagnosis at the time of her referral to plastic surgery and not discussed with the patient.

While most implant ruptures are clinically silent, some patients present with breast masses, pain, seroma formation, or silicone granuloma and adenopathy, as was the case in our patient. Pathologically, granuloma formation is mediated by histocytes and occurs as an inflammatory response to silicone leakage beyond the external capsule [13]. In patients with BIA-ALCL, seroma and mass represent the two most common phenotypes, cited to occur in approximately 80% and 10–20% of patients, respectively [14]. While the presence of implant rupture typically raises suspicion for a benign rather than malignant process, it is worth noting that according to the PROFILE registry, 13.5% of patient with BIA-ALCL had concurrent implant rupture, which likely occurred as a complication of the cancer [3]. Additionally, our patient had bilateral breast masses—an atypical finding in BIA-ALCL, cited to occur in only 2% of cases [3]. Retrospectively, this key feature of her case was more consistent with a diagnosis of silicone granuloma than BIA-ALCL.

As it pertains to imaging, mass enhancement with contrast MRI is much more typical of malignancy than silicone granuloma, though silicone enhancement has been reported in the literature and was observed in our patient [8]. Moreover, falsely positive PET scans occur with silicone granuloma and adenitis, in addition to other inflammatory states, given the high metabolic activity observed in inflammatory cells [8,15]. Carsen et al. described thirteen cases of silicone granuloma mimicking cancer. In their literature review, nine patients presented with breast masses, five patients had either axillary, mediastinal or internal mammary adenopathy, and four of the four patients who underwent PET imaging had false positive results [8]. Our patient’s imaging revealed generalized, enhancing adenopathy, an enhancing mass on MRI and marked left axillary radiotracer uptake, consistent with other cases of silicone granuloma mimicking cancer. While her left axillary adenopathy was not sampled, it was hypothesize to represent silicone adenitis.

Finally, the patient had small, bilateral peri-prosthetic effusions. While more than 80% of patient with BIA-ALCL present with seromas, the general incidence of unprovoked seromas forming more than 1 year after implantation is estimated to range from 0.88% to 1.84% [6]. While there is a lack of literature examining the possible causes of late seroma formation, a single case series of 60 late seromas published by Di Napoli A. found that only 9% were attributable to BIA-ALCL [10]. The other seromas were attributable to subclinical infection, hematoma, hypersensitivity reactions and implant leakage.

## Conclusion

ALCL is the most important current breast implant related issue. While clinicians and surgeons must be vigilant about prevention, diagnosis and treatment, it is important to remember that more common diagnoses are common so that we may accurately treat and counsel our patients who present with concerns about this disease process.
